# Ethyl 2-(4-nitro­benzamido)­benzoate, a non-merohedral twin

**DOI:** 10.1107/S1600536811002236

**Published:** 2011-01-22

**Authors:** Sohail Saeed, Naghmana Rashid, Jerry P. Jasinski, Ray J. Butcher

**Affiliations:** a Department of Chemistry, Research Complex, Allama Iqbal Open University, Islamabad 44000, Pakistan; b Department of Chemistry, Keene State College, 229 Main Street, Keene, NH 03435-2001, USA; c Department of Chemistry, Howard University, 525 College Street NW, Washington, DC 20059, USA

## Abstract

In the title compound, C_16_H_14_N_2_O_5_, a non-merohedral twin, the dihedral angle between the mean planes of the two benzene rings is 4.0 (9)°. The ethyl group is disordered [0.643 (14) and 0.357 (14) occupancy]. The nitro group is twisted by 16.4 (4)° from the mean plane of the benzene ring and the mean plane of the carbonyl group is twisted from the mean planes of the two benzene rings by 4.5 (0) and 4.7 (9)°. An intra­molecular N—H⋯O hydrogen bond occurs. The crystal packing is stabilized by weak inter­molecular C—H⋯O hydrogen-bond inter­actions.

## Related literature

For applications of amides and amide derivatives in the pharmaceutical industry, see: Banihashemi & Firoozifar (2003 ▶); Mallakpour & Kowsari (2005 ▶); Saxena *et al.* (2003 ▶); Wang *et al.* (2008 ▶). For standard bond lengths, see: Allen *et al.* (1987 ▶).
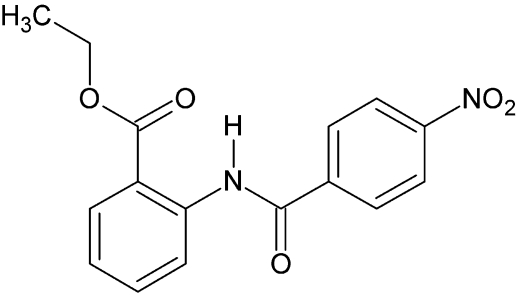


## Experimental

### 

#### Crystal data

C_16_H_14_N_2_O_5_
*M* *_r_* = 314.29
Triclinic, 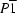

*a* = 6.9802 (3) Å
*b* = 9.3570 (4) Å
*c* = 12.5779 (5) Å
α = 102.833 (4)°
β = 94.296 (4)°
γ = 107.567 (4)°
*V* = 754.68 (6) Å^3^
*Z* = 2
Cu *K*α radiation
μ = 0.88 mm^−1^
*T* = 295 K
0.52 × 0.48 × 0.24 mm

#### Data collection

Oxford Diffraction Xcalibur Ruby Gemini diffractometer
Absorption correction: multi-scan (*CrysAlis RED*; Oxford Diffraction, 2007 ▶) *T*_min_ = 0.825, *T*_max_ = 1.000
10410 measured reflections
10410 independent reflections
9282 reflections with *I* > 2σ(*I*)

#### Refinement

*R*[*F* ^2^ > 2σ(*F* ^2^)] = 0.053
*wR*(*F* ^2^) = 0.157
*S* = 1.04
10410 reflections
218 parameters
25 restraints
H-atom parameters constrained
Δρ_max_ = 0.20 e Å^−3^
Δρ_min_ = −0.21 e Å^−3^

### 

Data collection: *CrysAlis PRO* (Oxford Diffraction, 2007 ▶); cell refinement: *CrysAlis RED* (Oxford Diffraction, 2007 ▶); data reduction: *CrysAlis RED*; program(s) used to solve structure: *SHELXS97* (Sheldrick, 2008 ▶); program(s) used to refine structure: *SHELXL97* (Sheldrick, 2008 ▶); molecular graphics: *SHELXTL* (Sheldrick, 2008 ▶); software used to prepare material for publication: *PLATON* (Spek, 2009 ▶).

## Supplementary Material

Crystal structure: contains datablocks global, I. DOI: 10.1107/S1600536811002236/hg2769sup1.cif

Structure factors: contains datablocks I. DOI: 10.1107/S1600536811002236/hg2769Isup2.hkl

Additional supplementary materials:  crystallographic information; 3D view; checkCIF report

## Figures and Tables

**Table 1:** Hydrogen-bond geometry (Å, °)

*D*—H⋯*A*	*D*—H	H⋯*A*	*D*⋯*A*	*D*—H⋯*A*
N2—H2⋯O4	0.86	1.95	2.6638 (9)	139
C2—H2*A*⋯O3^i^	0.93	2.50	3.4069 (11)	166
C10—H10*A*⋯O2^ii^	0.93	2.56	3.3716 (14)	146
C12—H12*A*⋯O1^iii^	0.93	2.50	3.2554 (12)	138

Symmetry codes: (i) 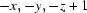; (ii) 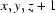; (iii) 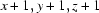.

**Fig. 1.**
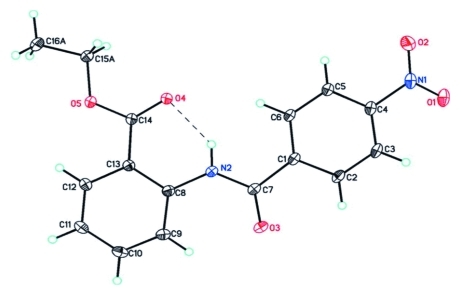
Molecular structure of C16H14N2O5, showing the atom labeling scheme and 50% probability displacement ellipsoids. Dashed lines indicate N—H···O intramolecular hydrogen bonding. C15A & C16A represent the major component (0.643 (14)) of the disordered ethyl group.

**Fig. 2.**
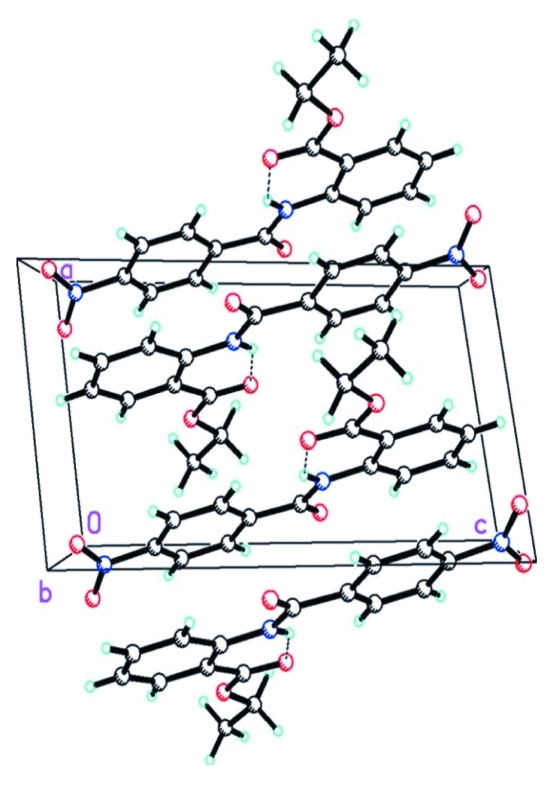
Packing diagram of the title compound viewed down the b axis. Dashed lined indicate N—H···O intramolecular hydrogen bonding.

## supplementary crystallographic information

### Comment

The development of heat-resistant, high performance polymers in the past decades has been quite dramatic and has drawn the attention of many polymer scientists all over the world. Wholly aromatic polymers such as polyamides and polyimides have already been noted for high temperature resistance and excellent physico-mechanical properties. Amides and amide derivatives have extensive applications in the pharmaceutical industry (Wang *et al.*, 2008) and in polymer chemistry (Saxena *et al.*, 2003; Banihashemi & Firoozifar, 2003; Mallakpour *et al.*, 2005).

In the title compound, C_16_H_14_N_2_O_5_, a nonmerohedral twin, the dihedral angle between the mean planes of the two benzene rings is 4.0 (9)° (Fig. 1). The ethyl group is disordered (0.643 (14) & 0.357 (14) occupancy). The nitro group is twisted by 16.4 (4)° from the mean plane of the benzene ring and the mean plane of the carbonyl group is twisted from the mean planes of the two benzene rings by 4.5 (0)° and 4.7 (9)°, respectively. Bond distances and angles are in normal ranges (Allen *et al.*, 1987). Crystal packing is stabilized by intramolecular N—H···O, and weak C—H···O intermolecular hydrogen bond interactions (Fig. 2).

### Experimental

A mixture of 4-nitrobenzoyl chloride (0.01 mol) and ethyl-*p*-aminobenzoate (0.01 mol) was refluxed in anhydrous acetone (70 ml) for three hours. After cooling to room temperature, the mixture was poured into acidified cold water. The resulting yellow solid product was filtered and washed with cold acetone. Single crystals of the title compound suitable for single-crystal *x*-ray analysis were obtained by recrystallization of the yellow solid from ethyl acetate.

### Refinement

This structure has been refined as a nonmerohedral twin and the nonmerohedral twin matrix has been identified. The ethyl group carbon atoms are disordered with occupancies 0.643 (14) (C15A & C16A) and 0.357 (14) (C15B & C16B), respectively. All of the other H atoms were placed in their calculated positions and then refined using the riding model with Atom–H lengths of 0.93Å (CH), 0.97Å (CH_2_), or 0.96Å (CH_3_) or 0.86Å (NH). Isotropic displacement parameters for these atoms were set to 1.19–1.20 (CH), 1.20 (CH_2_), 1.49 (CH_3_) or 1.20 (NH) times *U*_eq_ of the parent atom.

### Crystal data

**Table:**

C_16_H_14_N_2_O_5_	*Z* = 2
*M**_r_* = 314.29	*F*(000) = 328
Triclinic, *P*1	*D*_x_ = 1.383 Mg m^−^^3^
Hall symbol: -P 1	Cu *K*α radiation, λ = 1.54178 Å
*a* = 6.9802 (3) Å	Cell parameters from 4228 reflections
*b* = 9.3570 (4) Å	θ = 5.1–73.9°
*c* = 12.5779 (5) Å	µ = 0.88 mm^−^^1^
α = 102.833 (4)°	*T* = 295 K
β = 94.296 (4)°	Block, yellow
γ = 107.567 (4)°	0.52 × 0.48 × 0.24 mm
*V* = 754.68 (6) Å^3^	

### Data collection

**Table:**

Oxford Diffraction Xcalibur Ruby Gemini diffractometer	10410 measured reflections
Radiation source: Enhance (Cu) X-ray Source	10410 independent reflections
graphite	9282 reflections with *I* > 2σ(*I*)
Detector resolution: 10.5081 pixels mm^-1^	θ_max_ = 74.5°, θ_min_ = 5.1°
ω scans	*h* = −8→8
Absorption correction: multi-scan (*CrysAlis RED*; Oxford Diffraction, 2007)	*k* = −11→11
*T*_min_ = 0.825, *T*_max_ = 1.000	*l* = −15→15

### Refinement

**Table:**

Refinement on *F*^2^	Primary atom site location: structure-invariant direct methods
Least-squares matrix: full	Secondary atom site location: difference Fourier map
*R*[*F*^2^ > 2σ(*F*^2^)] = 0.053	Hydrogen site location: inferred from neighbouring sites
*wR*(*F*^2^) = 0.157	H-atom parameters constrained
*S* = 1.04	*w* = 1/[σ^2^(*F*_o_^2^) + (0.0935*P*)^2^ + 0.0632*P*] where *P* = (*F*_o_^2^ + 2*F*_c_^2^)/3
10410 reflections	(Δ/σ)_max_ < 0.001
218 parameters	Δρ_max_ = 0.20 e Å^−^^3^
25 restraints	Δρ_min_ = −0.21 e Å^−^^3^

### Special details

**Table:**

Geometry. All e.s.d.'s (except the e.s.d. in the dihedral angle between two l.s. planes) are estimated using the full covariance matrix. The cell e.s.d.'s are taken into account individually in the estimation of e.s.d.'s in distances, angles and torsion angles; correlations between e.s.d.'s in cell parameters are only used when they are defined by crystal symmetry. An approximate (isotropic) treatment of cell e.s.d.'s is used for estimating e.s.d.'s involving l.s. planes.
Refinement. Refinement of *F*^2^ against ALL reflections. The weighted *R*-factor *wR* and goodness of fit *S* are based on *F*^2^, conventional *R*-factors *R* are based on *F*, with *F* set to zero for negative *F*^2^. The threshold expression of *F*^2^ > σ(*F*^2^) is used only for calculating *R*-factors(gt) *etc*. and is not relevant to the choice of reflections for refinement. *R*-factors based on *F*^2^ are statistically about twice as large as those based on *F*, and *R*- factors based on ALL data will be even larger.

### Fractional atomic coordinates and isotropic or equivalent isotropic displacement parameters (Å^2^)

**Table:**

	*x*	*y*	*z*	*U*_iso_*/*U*_eq_	Occ. (<1)
O1	−0.18667 (13)	0.04273 (9)	0.01289 (7)	0.1014 (3)	
O2	0.02489 (16)	0.25940 (11)	0.00650 (7)	0.1088 (3)	
O3	0.12371 (18)	0.20254 (8)	0.56742 (8)	0.1132 (3)	
O4	0.40086 (11)	0.74605 (7)	0.55727 (5)	0.06973 (19)	
O5	0.52758 (10)	0.93554 (6)	0.71099 (5)	0.06630 (18)	
N1	−0.05477 (14)	0.16683 (10)	0.05611 (8)	0.0793 (3)	
N2	0.24927 (11)	0.45878 (8)	0.57997 (6)	0.06052 (19)	
H2	0.2733	0.5241	0.5401	0.073*	
C1	0.11370 (12)	0.27848 (8)	0.40099 (8)	0.0583 (2)	
C2	0.01013 (15)	0.12590 (9)	0.34180 (9)	0.0697 (3)	
H2A	−0.0232	0.0483	0.3789	0.084*	
C3	−0.04350 (15)	0.08854 (10)	0.22924 (9)	0.0725 (3)	
H3A	−0.1131	−0.0134	0.1900	0.087*	
C4	0.00737 (13)	0.20403 (10)	0.17595 (8)	0.0637 (2)	
C5	0.11309 (14)	0.35587 (10)	0.23110 (8)	0.0653 (2)	
H5A	0.1488	0.4323	0.1930	0.078*	
C6	0.16450 (14)	0.39178 (9)	0.34375 (8)	0.0634 (2)	
H6A	0.2347	0.4940	0.3822	0.076*	
C7	0.16219 (14)	0.30877 (9)	0.52350 (8)	0.0657 (2)	
C8	0.30613 (12)	0.52356 (9)	0.69344 (7)	0.0574 (2)	
C9	0.26962 (16)	0.43559 (12)	0.77040 (9)	0.0753 (3)	
H9A	0.2068	0.3287	0.7464	0.090*	
C10	0.32558 (18)	0.50539 (14)	0.88137 (10)	0.0870 (3)	
H10A	0.2980	0.4450	0.9315	0.104*	
C11	0.42132 (18)	0.66243 (14)	0.91963 (9)	0.0817 (3)	
H11A	0.4610	0.7079	0.9949	0.098*	
C12	0.45786 (14)	0.75151 (11)	0.84562 (8)	0.0656 (2)	
H12A	0.5215	0.8581	0.8714	0.079*	
C13	0.40165 (12)	0.68549 (9)	0.73274 (7)	0.0541 (2)	
C14	0.44109 (12)	0.78829 (9)	0.65684 (7)	0.0544 (2)	
C15A	0.5896 (13)	1.0482 (9)	0.6489 (8)	0.0698 (11)	0.643 (14)
H15A	0.4716	1.0511	0.6046	0.084*	0.643 (14)
H15B	0.6805	1.0202	0.5997	0.084*	0.643 (14)
C16A	0.6954 (13)	1.2037 (5)	0.7264 (5)	0.0928 (12)	0.643 (14)
H16A	0.7493	1.2776	0.6850	0.139*	0.643 (14)
H16B	0.8043	1.1978	0.7747	0.139*	0.643 (14)
H16C	0.6005	1.2359	0.7693	0.139*	0.643 (14)
C15B	0.550 (2)	1.0505 (18)	0.6437 (16)	0.0698 (11)	0.357 (14)
H15C	0.6508	1.0448	0.5953	0.084*	0.357 (14)
H15D	0.4216	1.0358	0.6001	0.084*	0.357 (14)
C16B	0.6185 (17)	1.1998 (11)	0.7309 (9)	0.0928 (12)	0.357 (14)
H16D	0.6536	1.2847	0.6970	0.139*	0.357 (14)
H16E	0.7352	1.2045	0.7788	0.139*	0.357 (14)
H16F	0.5108	1.2062	0.7729	0.139*	0.357 (14)

### Atomic displacement parameters (Å^2^)

**Table:**

	*U*^11^	*U*^22^	*U*^33^	*U*^12^	*U*^13^	*U*^23^
O1	0.0958 (5)	0.0834 (5)	0.0877 (5)	0.0130 (4)	−0.0132 (4)	−0.0212 (4)
O2	0.1291 (7)	0.1025 (6)	0.0697 (5)	0.0111 (5)	0.0051 (5)	0.0127 (5)
O3	0.1821 (9)	0.0505 (4)	0.0854 (5)	0.0055 (4)	0.0053 (5)	0.0245 (4)
O4	0.0973 (5)	0.0501 (3)	0.0505 (3)	0.0120 (3)	0.0030 (3)	0.0100 (2)
O5	0.0821 (4)	0.0482 (3)	0.0557 (3)	0.0088 (3)	0.0018 (3)	0.0079 (3)
N1	0.0766 (5)	0.0723 (5)	0.0714 (5)	0.0212 (4)	0.0004 (4)	−0.0088 (4)
N2	0.0725 (4)	0.0454 (3)	0.0590 (4)	0.0133 (3)	0.0046 (3)	0.0141 (3)
C1	0.0556 (4)	0.0421 (4)	0.0717 (5)	0.0131 (3)	0.0071 (4)	0.0085 (4)
C2	0.0723 (6)	0.0429 (4)	0.0849 (7)	0.0114 (4)	0.0083 (5)	0.0103 (4)
C3	0.0699 (5)	0.0444 (4)	0.0846 (7)	0.0095 (4)	0.0019 (5)	−0.0045 (4)
C4	0.0564 (5)	0.0568 (5)	0.0677 (5)	0.0178 (4)	0.0045 (4)	−0.0015 (4)
C5	0.0690 (5)	0.0524 (4)	0.0646 (5)	0.0122 (4)	0.0055 (4)	0.0075 (4)
C6	0.0684 (5)	0.0415 (4)	0.0670 (5)	0.0073 (3)	0.0039 (4)	0.0048 (3)
C7	0.0742 (5)	0.0443 (4)	0.0740 (6)	0.0128 (4)	0.0091 (4)	0.0159 (4)
C8	0.0570 (4)	0.0561 (4)	0.0608 (5)	0.0187 (3)	0.0059 (4)	0.0190 (4)
C9	0.0854 (6)	0.0668 (5)	0.0742 (6)	0.0189 (5)	0.0047 (5)	0.0303 (5)
C10	0.1013 (8)	0.0948 (8)	0.0730 (7)	0.0277 (6)	0.0080 (5)	0.0454 (6)
C11	0.0959 (7)	0.0933 (7)	0.0550 (5)	0.0294 (6)	−0.0004 (5)	0.0236 (5)
C12	0.0678 (5)	0.0696 (5)	0.0558 (5)	0.0210 (4)	0.0006 (4)	0.0137 (4)
C13	0.0517 (4)	0.0554 (4)	0.0550 (4)	0.0185 (3)	0.0043 (3)	0.0137 (3)
C14	0.0551 (4)	0.0501 (4)	0.0532 (4)	0.0144 (3)	0.0038 (3)	0.0092 (3)
C15A	0.084 (3)	0.0519 (5)	0.0668 (10)	0.0134 (15)	0.0017 (19)	0.0182 (6)
C16A	0.114 (3)	0.0528 (6)	0.0912 (10)	0.0040 (19)	0.005 (2)	0.0139 (6)
C15B	0.084 (3)	0.0519 (5)	0.0668 (10)	0.0134 (15)	0.0017 (19)	0.0182 (6)
C16B	0.114 (3)	0.0528 (6)	0.0912 (10)	0.0040 (19)	0.005 (2)	0.0139 (6)

### Geometric parameters (Å, °)

**Table:**

O1—N1	1.2210 (11)	C8—C13	1.4116 (12)
O2—N1	1.2042 (12)	C9—C10	1.3735 (16)
O3—C7	1.2126 (11)	C9—H9A	0.9300
O4—C14	1.2099 (10)	C10—C11	1.3720 (17)
O5—C14	1.3214 (10)	C10—H10A	0.9300
O5—C15A	1.431 (12)	C11—C12	1.3705 (14)
O5—C15B	1.49 (2)	C11—H11A	0.9300
N1—C4	1.4694 (13)	C12—C13	1.3899 (13)
N2—C7	1.3497 (11)	C12—H12A	0.9300
N2—C8	1.3955 (12)	C13—C14	1.4833 (12)
N2—H2	0.8600	C15A—C16A	1.492 (6)
C1—C6	1.3851 (12)	C15A—H15A	0.9700
C1—C2	1.3926 (12)	C15A—H15B	0.9700
C1—C7	1.4963 (13)	C16A—H16A	0.9600
C2—C3	1.3742 (15)	C16A—H16B	0.9600
C2—H2A	0.9300	C16A—H16C	0.9600
C3—C4	1.3670 (14)	C15B—C16B	1.490 (12)
C3—H3A	0.9300	C15B—H15C	0.9700
C4—C5	1.3773 (12)	C15B—H15D	0.9700
C5—C6	1.3743 (14)	C16B—H16D	0.9600
C5—H5A	0.9300	C16B—H16E	0.9600
C6—H6A	0.9300	C16B—H16F	0.9600
C8—C9	1.3943 (13)		
			
C14—O5—C15A	118.5 (3)	C8—C9—H9A	119.7
C14—O5—C15B	116.1 (5)	C11—C10—C9	121.27 (9)
O2—N1—O1	123.88 (10)	C11—C10—H10A	119.4
O2—N1—C4	118.86 (8)	C9—C10—H10A	119.4
O1—N1—C4	117.26 (10)	C12—C11—C10	119.23 (10)
C7—N2—C8	129.57 (7)	C12—C11—H11A	120.4
C7—N2—H2	115.2	C10—C11—H11A	120.4
C8—N2—H2	115.2	C11—C12—C13	121.24 (9)
C6—C1—C2	118.55 (9)	C11—C12—H12A	119.4
C6—C1—C7	124.29 (7)	C13—C12—H12A	119.4
C2—C1—C7	117.16 (8)	C12—C13—C8	119.45 (8)
C3—C2—C1	120.87 (9)	C12—C13—C14	118.76 (8)
C3—C2—H2A	119.6	C8—C13—C14	121.78 (7)
C1—C2—H2A	119.6	O4—C14—O5	122.72 (7)
C4—C3—C2	118.78 (8)	O4—C14—C13	125.49 (7)
C4—C3—H3A	120.6	O5—C14—C13	111.79 (7)
C2—C3—H3A	120.6	O5—C15A—C16A	109.2 (7)
C3—C4—C5	122.19 (9)	O5—C15A—H15A	109.8
C3—C4—N1	119.44 (8)	C16A—C15A—H15A	109.8
C5—C4—N1	118.36 (9)	O5—C15A—H15B	109.8
C6—C5—C4	118.41 (8)	C16A—C15A—H15B	109.8
C6—C5—H5A	120.8	H15A—C15A—H15B	108.3
C4—C5—H5A	120.8	C16B—C15B—O5	101.6 (13)
C5—C6—C1	121.18 (8)	C16B—C15B—H15C	111.4
C5—C6—H6A	119.4	O5—C15B—H15C	111.4
C1—C6—H6A	119.4	C16B—C15B—H15D	111.4
O3—C7—N2	123.20 (9)	O5—C15B—H15D	111.4
O3—C7—C1	120.67 (8)	H15C—C15B—H15D	109.3
N2—C7—C1	116.13 (7)	C15B—C16B—H16D	109.5
C9—C8—N2	122.86 (8)	C15B—C16B—H16E	109.5
C9—C8—C13	118.22 (8)	H16D—C16B—H16E	109.5
N2—C8—C13	118.91 (7)	C15B—C16B—H16F	109.5
C10—C9—C8	120.58 (9)	H16D—C16B—H16F	109.5
C10—C9—H9A	119.7	H16E—C16B—H16F	109.5
			
C6—C1—C2—C3	−1.00 (14)	C13—C8—C9—C10	−0.03 (15)
C7—C1—C2—C3	178.82 (9)	C8—C9—C10—C11	−1.01 (18)
C1—C2—C3—C4	0.25 (15)	C9—C10—C11—C12	1.36 (18)
C2—C3—C4—C5	1.01 (15)	C10—C11—C12—C13	−0.66 (17)
C2—C3—C4—N1	−177.99 (8)	C11—C12—C13—C8	−0.36 (14)
O2—N1—C4—C3	−164.54 (10)	C11—C12—C13—C14	178.66 (9)
O1—N1—C4—C3	15.94 (13)	C9—C8—C13—C12	0.70 (13)
O2—N1—C4—C5	16.42 (14)	N2—C8—C13—C12	179.60 (8)
O1—N1—C4—C5	−163.10 (9)	C9—C8—C13—C14	−178.29 (8)
C3—C4—C5—C6	−1.46 (15)	N2—C8—C13—C14	0.62 (12)
N1—C4—C5—C6	177.55 (8)	C15A—O5—C14—O4	−4.7 (4)
C4—C5—C6—C1	0.66 (14)	C15B—O5—C14—O4	7.5 (7)
C2—C1—C6—C5	0.53 (14)	C15A—O5—C14—C13	175.3 (4)
C7—C1—C6—C5	−179.27 (8)	C15B—O5—C14—C13	−172.6 (7)
C8—N2—C7—O3	−1.98 (17)	C12—C13—C14—O4	179.61 (8)
C8—N2—C7—C1	177.96 (8)	C8—C13—C14—O4	−1.39 (14)
C6—C1—C7—O3	−175.60 (10)	C12—C13—C14—O5	−0.37 (11)
C2—C1—C7—O3	4.60 (15)	C8—C13—C14—O5	178.63 (7)
C6—C1—C7—N2	4.46 (14)	C14—O5—C15A—C16A	−176.2 (3)
C2—C1—C7—N2	−175.34 (8)	C15B—O5—C15A—C16A	104 (5)
C7—N2—C8—C9	−3.14 (15)	C14—O5—C15B—C16B	171.2 (6)
C7—N2—C8—C13	178.00 (8)	C15A—O5—C15B—C16B	−83 (4)
N2—C8—C9—C10	−178.89 (10)		

### Hydrogen-bond geometry (Å, °)

**Table:**

*D*—H···*A*	*D*—H	H···*A*	*D*···*A*	*D*—H···*A*
N2—H2···O4	0.86	1.95	2.6638 (9)	139
C2—H2A···O3^i^	0.93	2.50	3.4069 (11)	166
C10—H10A···O2^ii^	0.93	2.56	3.3716 (14)	146
C12—H12A···O1^iii^	0.93	2.50	3.2554 (12)	138

Symmetry codes: (i) −*x*, −*y*, −*z*+1; (ii) *x*, *y*, *z*+1; (iii) *x*+1, *y*+1, *z*+1.
